# Nonlinear Dielectric Spectroscopy as an Indirect Probe of Metabolic Activity in Thylakoid Membrane

**DOI:** 10.3390/bios1010013

**Published:** 2011-01-31

**Authors:** Jie Fang, Akilan Palanisami, Kimal Rajapakshe, William R. Widger, John H. Miller

**Affiliations:** 1 Department of Physics and Texas Center for Superconductivity, University of Houston, Houston, TX 77204, USA; E-Mails: apalanisami@uh.edu (A.P.); kirajapa@mail.uh.edu (K.R.); jhmiller@uh.edu (J.H.M.); 2 Department of Biology and Biochemistry, University of Houston, Houston, TX 77204, USA; E-Mail: widger@uh.edu (W.R.W.)

**Keywords:** nonlinear dielectric spectroscopy, reactive oxygen species, whole cell biosensor, thylakoid

## Abstract

Nonlinear dielectric spectroscopy (NDS) is a non-invasive probe of cellular metabolic activity with potential application in the development of whole-cell biosensors. However, the mechanism of NDS interaction with metabolic membrane proteins is poorly understood, partly due to the inherent complexity of single cell organisms. Here we use the light-activated electron transport chain of spinach thylakoid membrane as a model system to study how NDS interacts with metabolic activity. We find protein modification, as opposed to membrane pump activity, to be the dominant source of NDS signal change in this system. Potential mechanisms for such protein modifications include reactive oxygen species generation and light-activated phosphorylation.

## 1. Introduction

The most commonly studied biosensors typically use purified enzymes and antibodies for detection. While displaying a high degree of specificity, these assays have some drawbacks including unwanted sensitivity to experimental conditions, lengthy preparation time and high cost. Compared to enzymes and antibodies, biosensors using single cell microorganisms have several singular advantages, one being microorganisms have evolved to function in a variety of environments. This allows sensing in regimes that would denature ordinary molecular biosensors. In addition, microorganisms reproduce rapidly and require little in the way of preparation, as opposed to antibody or enzyme techniques, which require time consuming purification. Another advantage of using whole cells as the biosensing element is that they can be used as a broad spectrum indicator of a sample’s toxicity. In particular, whole cell biosensors can analyze samples via multi-step enzymatic processes, such as respiration or fermentation. The impact of the analyte on these processes can then be evaluated for the physiological impact on higher level organisms (e.g., humans). 

In whole cell biosensors, suspended cells are exposed to the analyte, and the signal read out via the cellular response. The readout used depends on the mechanism by which the analyte interacts with cell, and a number of readout methods have been developed including O_2_ consumption [1,2], pH change [3], impedance spectroscopy [4,5,6], and luminescence [7,8,9]. A limiting factor in whole cell biosensors is the degree of information which can be extracted via these readout methods. If the readout methods could probe in more detail the effects of the analyte on the cell, more information could be gleaned about the nature of the analyte. In this vein, nonlinear dielectric spectroscopy (NDS) is a promising method that can noninvasively probe cellular behavior by monitoring the electrical response of a cellular suspension to an electric field. In the low frequency domain (less than 10 kHz), the nonlinear response probes the local free energy landscape of membrane dipole moments. If this free energy landscape changes (perhaps due to changes in dipole moment or ligand binding), the nonlinear response will also change [10], allowing NDS to be used as a sensitive probe of membrane protein behavior. In nature, membrane proteins play a large role in the metabolic electron transport chain (ETC), and NDS could be a convenient, non-invasive probe of such metabolic activity. Prior work has found clear evidence that NDS is sensitive to metabolic activity [11,12,13,14]. However, the mechanisms of metabolic activity probed by NDS are poorly characterized and require further study.

In this study, we use the thylakoid membrane from spinach as a model system to examine this question. Thylakoid membranes are similar to mitochondria and bacteria since all three use electron transport processes to generate a proton motive force [15] (essentially a voltage and/or proton gradient), but are much less complex. Another advantage of the thylakoid membrane system is the ETC is driven by photosynthesis and can be easily activated by light. In addition, the proton motive force and ETC can both be influenced by various inhibitors or uncouplers as well as chemicals which enhance the production of reactive oxygen species (ROS). These properties of the thylakoid membrane suspension allow a more detailed study of the mechanisms of metabolism that contributes to the nonlinear dielectric response.

## 2. Experimental Section

Spinach leaf thylakoids were isolated by differential centrifugation [16]. 300 mL loosely packed fresh spinach leaves were purchased from a grocery store and blended on high speed for 15 s with a blender in 300 mL 0.3 M NaCl, 30 mM Tricine, 3 mM MgCl_2_ and 0.5 mM EDTA, pH 7.8 (grinding buffer). The suspension was then filtered through 8 layers of cheese cloth, and the filtrate centrifuged at 3,000 ×*g* for 4 min. The pellet was resuspended in 0.2 M sucrose, 5 mM HEPES, 2 mM MgCl_2_ and 0.05% bovine serum albumin, pH 7.5 (suspension buffer). The suspension was then centrifuged at 1,600 ×*g* for 30 s to remove cell debris. Next, the supernatant was filtered through a kim wipe. The filtrate was again centrifuged at 3,000 ×*g* for 4 min, and the supernatant discarded. The pellet was then resuspended in suspension buffer to the desired concentration. All procedures were performed at 4 °C. Thylakoid concentration was measured via the absorbance at 562 nm to determine the chlorophyll concentration [16]. All measurements were done within 3 hours of thylakoid isolation.

**Figure 1:**
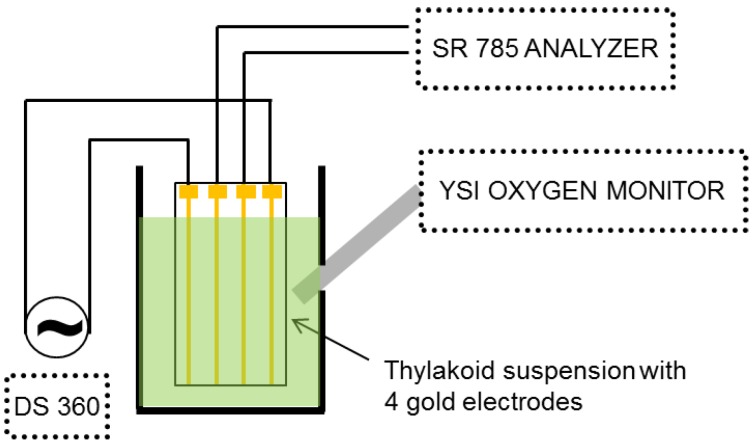
Four-probe measurement setup used to measure nonlinear dielectric spectrum and oxygen concentration (oxygen sensor not shown).

An external electric field was applied to the sample using a Stanford Research DS 360 low distortion function generator (Figure 1). A four-terminal gold electrode system [11] was used to apply the voltage and detect the harmonics generated in the thylakoid suspension. The outer two electrodes were used to provide the electric field to the thylakoid suspension, while the response was monitored by measuring the voltage across the inner two electrodes. To probe the nonlinearity, the harmonic response of the inner electrodes was measured with a Stanford Research SR 785 FFT spectrum analyzer. Because a pure sinusoid was used for the driving voltage, the frequency response consists of the driving frequency (f) and its harmonics (2f, 3f, *etc*.). To ensure that the observed harmonics were only due to the thylakoid suspension, the sinusoidal electric field provided by the DS 360 was measured for linearity and found to have better than −100 dB distortion over the audio frequency range. Gold wire used for the electrodes had a diameter of 0.01 cm (0.004 inch), and the distance between each electrode was 0.23 cm. The wires were mounted in an open plastic frame which held the wires taut, and the length of the wire electrodes exposed to the suspension was 0.5 cm. The measurement chamber was mixed by magnetic stirring. During NDS measurements, oxygen concentrations were simultaneously measured with a YSI oxygen electrode. All the data were analyzed by LabView (National Instruments).

K_3_Fe(CN)_6_ (1.0 mM) or methyl viologen (MV; 100 μM) with sodium azide (1 mM) were used as terminal electron acceptors. All experiments began with measurement in the dark state, ensuring a non-active ETC. The thylakoid suspension was then subjected to light from a 320 W projection lamp (General Electric DAY/DAK) for 2 min. This light was filtered via a 10 cm round bottom flask filled with saturated copper sulfate (CuSO_4_) to avoid heating the thylakoid suspension. This system also allowed the light to be focused on the sample. When performing fixed frequency experiments, the applied electric field was fixed at 3,072 Hz with amplitude of 8 V_pp_.

The nonlinear response was also measured in the presence or absence of 20 μM 1-dimethylurea (DCMU), an inhibitor of photosystem II. Additional measurements were performed in the presence or absence of the uncouplers ammonium chloride (NH_4_Cl; 2 mM) or gramicidin (21.2 μM). 

## 3. Results and Discussion

**Figure 2:**
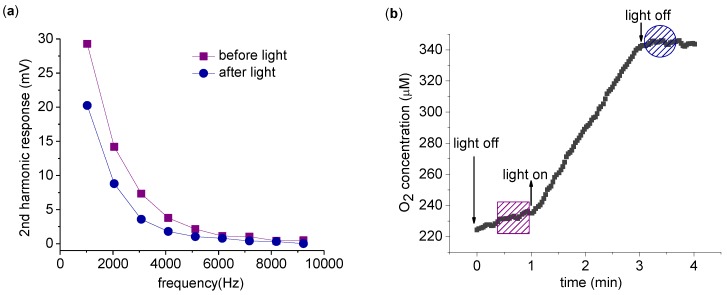
Measurement of the 2nd harmonic response with varying driving frequency. **(a)** 2nd harmonic response before (square) and immediately after (circle) a 2 min exposure to light. Both 2nd harmonic response measurements are taken in the dark, with each point the average of a 4 s acquisition and 8 V_pp_ driving voltage. **(b)** O_2_ concentration was recorded at all times. The time span of harmonic measurement is indicated by a box (before light application) and a circle (after light application). The measurement was carried out with 0.2 mg/mL chlorophyll in 5 mL suspension buffer with 1.0 mM K_3_Fe(CN)_6_ as described in the text.

The harmonics produced by spinach thylakoid in response to AC electric fields at frequencies between 1 kHz and 10 kHz were examined at a fixed amplitude of 8 V_pp_. Measurements in the dark were compared before and after a 2 min application of light (Figure 2(a)). As seen in Figure 2(a), the 2nd harmonic response decreases after light exposure. To confirm the photosynthetic activation of the ETC, the buffer oxygen concentration was measured simultaneously with the harmonic measurements (Figure 2(b)). The evolution of O_2_ is clear evidence of photosynthetic ETC activity. 

**Figure 3:**
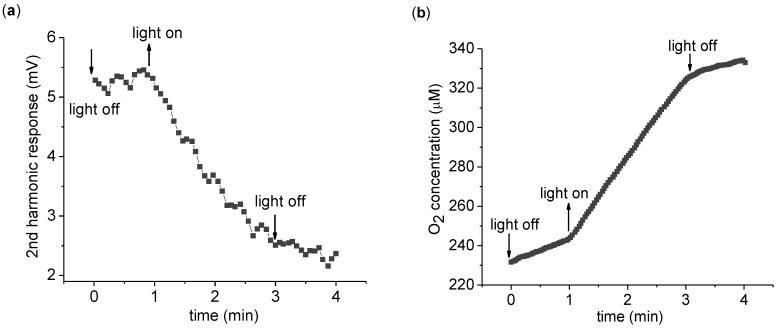
Continuous measurement of the 2nd harmonic response. **(a)** Time dependence of the 2nd harmonic response with a 3,072 Hz, 8 Vpp driving sinusoidal voltage. Every point is a 4 s average of the 2nd harmonic response. **(b)** The measurement of the O_2_ concentration of the suspension was taken simultaneously with the 2nd harmonic response seen in (a). The measurement was carried out with 0.2 mg/mL chlorophyll in 5 mL suspension buffer with 1.0 mM K_3_Fe(CN)_6_ as described in the text. The O_2_ concentration increases in the dark due to O_2_ absorption from the atmosphere.

The time dependence of the 2nd harmonic response is more explicitly monitored in Figure 3. Upon light exposure, the 2nd harmonic response is seen to gradually reduce in magnitude. A key point here is the irreversible nature of the change—the 2nd harmonic response does not return to its original value after the light is turned off. Thus the 2nd harmonic response is not directly probing ETC activity, but some related aspect. To measure the change in the 2nd harmonic response, a convenient measure is the difference of the 2nd harmonic response before and after light application. To this end, we measure the average 2nd harmonic response over 1 min immediately before (V_before_) and after (V_after_) 2 min of light application. We define the 2nd harmonic difference as

*∆V_2f_* = *V_after_* − *V_before_* (1)

**Figure 4:**
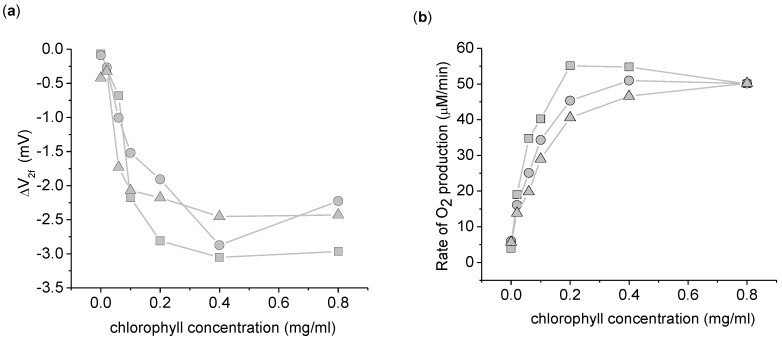
Dependence of the 2nd harmonic response to thylakoid concentration. **(a)** ΔV_2f_ taken with a 3,072 Hz, 8 V_pp_ sinusoid driving voltage. **(b)** Rate of increase in O_2_ concentration. This data was taken simultaneously with ΔV_2f_. This experiment was done in triplicate, with each symbol (circle, square, and triangle) representing a different replicate per chlorophyll concentration. The background O_2_ absorption from the atmosphere was corrected for in this data by measuring the rate of O_2_ increase of the buffer in the dark for 1 min before and after the light exposure. The background O_2_ absorption was taken to be the average of the two dark measurements. Measurements use 5 mL suspension buffer with 1.0 mM K_3_Fe(CN)_6_.

To confirm that the origin of the ΔV_2f_ was biological in nature and not due to light induced surface chemistry at the electrodes, the dependence of ΔV_2f_ with thylakoid concentration was investigated (Figure 4). A clear dependence of ΔV_2f_ on thylakoid concentration is apparent, ruling out electrode-light interactions. Beyond a thylakoid concentration of 0.2 mg/mL chlorophyll, ΔV_2f_ and the O_2_ production are both relatively independent of the thylakoid concentration. At these higher concentrations, the thylakoids become light limited. In the other experiments reported here, we chose 0.2 mg/mL as a compromise between maximizing ΔV_2f_ and ensuring light saturation of the thylakoids.

To determine whether the ΔV_2f_ was correlated with O_2_ production, K_3_Fe(CN)_6_ was replaced with 100 µM MV + 1 mM sodium azide. Similar with K_3_Fe(CN)_6_, MV can also accept electrons from the ETC. However, unlike K_3_Fe(CN)_6_, these electrons are quickly transferred to molecular oxygen creating superoxide (O_2_^−^), which subsequently dismutase into peroxide (O_2_^2−^) and oxygen. The net reaction during photosynthesis is to remove molecular dioxygen from the system, as shown by the equation. 


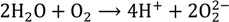
(2)

**Figure 5:**
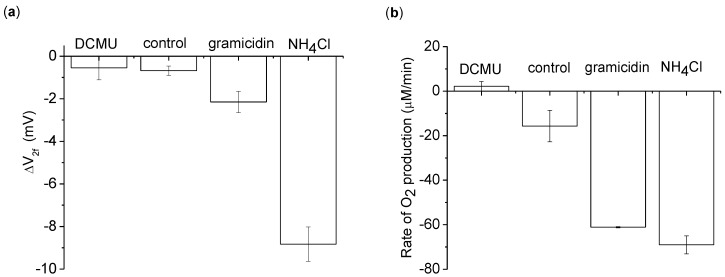
The 2nd harmonic response using 100 µM methyl viologen (MV) + 1 mM sodium azide as an electron acceptor. Measurements are of a 5 mL thylakoid suspension with 0.2 mg/mL chlorophyll. Additional conditions are given in different columns: 20 µM 1-dimethylurea (DCMU), 2 mM NH_4_Cl, and 21.2 µM gramicidin. **(a)** ΔV_2f_ measured with a 3,072 Hz, 8 V_pp_ sinusoid driving voltage. **(b)** The average O_2_ production rate during the 2 min light application. This data was measured simultaneously with ΔV_2f_. The background O_2_ absorption from the atmosphere was corrected for in this data by measuring the rate of O_2_ increase of the buffer in the dark for 1 min before and after the light exposure. The background O_2_ absorption was taken to be the average of the two dark measurements. Each measurement was done 3 times, with the average and standard deviation shown. The one exception was in the O_2_ data of gramicidin, where one measurement failed due to operator error and was removed from the analysis.

Sodium azide is added to inhibit the enzyme catalase, which catalyzes the decomposition of peroxide to water and oxygen [17]. From the “control” column of Figure 5, we see that even with MV+azide creating a decrease in O_2_ (Figure 5(b)), a negative ΔV_2f_ is still apparent [Figure 5(a)]. We conclude that ΔV_2f_ is not dependent on O_2_ concentration

MV+azide experiments exhibited a reduced ΔV_2f_ magnitude but an increased reproducibility of the NDS response as compared to K_3_Fe(CN)_6_. Apparently, the [Fe(CN)_6_]^3−^ complex and its reduced counterpart [Fe(CN)_6_]^4−^ can interact electrochemically with the electrodes under these conditions, increasing the run-to-run uncertainty within the system (the ΔV_2f_ standard deviation roughly doubled in the K_3_Fe(CN)_6_ system as compared to the MV+azide system). With this in mind, we performed the subsequent experiments using MV+azide as the electron acceptor.

To determine what aspect of ETC operation was contributing to ΔV_2f_, ETC activity was modulated chemically by adding the inhibitor DCMU, or the uncouplers gramicidin or NH_4_Cl. DCMU blocks the ETC from passing electrons between photosystem II to photosystem I. With the ETC blocked, no statistically significant change in ΔV_2f_ is apparent after light application [Figure 5(a)]. 

On the other hand, the uncouplers gramicidin or NH_4_Cl increase ETC activity by making the thylakoid membrane permeable to protons. Under normal thylakoid operation, a pH gradient/membrane potential builds up and opposes ETC operation. By adding an uncoupler, the pH gradient/membrane potential is dissipated, and the ETC can operate unconstrained. This increase in activity due to the uncoupling can be seen in the O_2_ concentration behavior [Figure 5(b)]. In particular, adding gramicidin increases both the ETC activity and ΔV_2f_ by roughly a factor of 4, whereas NH_4_Cl increases ΔV_2f_ by a substantially larger amount (Table 1).

**Table 1:** The ratio of ΔV_2f_ to O_2_ production (from data in Figure 5). The control and gramicidin entries are similar, suggesting ΔV_2f_ scales linearly with O_2_ production under gramicidin induced uncoupling. The NH_4_Cl ratio is significantly larger than the gramicidin ratio. Error is given as standard deviation.

	ΔV_2f_/O_2_ production (mV min/μM)
control	0.047 ± 0.048
gramicidin	0.070 ± 0.016
NH_4_Cl	0.256 ± 0.028

One potential mechanism for these changes in ΔV_2f_ is the production of ROS, which can modify membrane proteins. Even without MV, a natural byproduct of photosynthetic ETC is the production of superoxide. The rate of superoxide production is found to correlate with the rate of ETC activity [18]. Superoxide can breakdown into particularly damaging compounds such as peroxynitrite and the very reactive hydroxyl radical [19]. These toxins react with many biological molecules in a variety of mechanisms [20,21] and will, in many cases, alter local dipole moments. Such dipole moment alterations are expected to be focused near the thylakoid membrane, and since low frequency NDS couples especially strongly to dipoles associated with lipid membranes [10], it is expected that such ROS induced dipole moment alterations would appear in NDS measurements. 

Increased ETC activity can thereby create membrane localized dipole moment alterations. Gramicidin increases the ETC rate, therefore increasing ROS production [18]. However, NH_4_Cl not only increases the ETC rate, but can also actively promote greater ROS damage [22], which can further increase the NDS response. This mechanism could explain why ΔV_2f_ is larger in magnitude for NH_4_Cl treated thylakoid membranes as compared to the gramicidin treated condition, despite exhibiting similar rates of ETC activity (as judging from the O_2_ consumption).

Whatever ROS induced dipole moment alterations are occurring, it does not strongly affect the ETC activity, as the ETC activity is constant during photosynthesis [Figure 3(b)]. This is expected as chloroplasts have developed natural defenses against ROS. For instance, thylakoid membranes are known to be rich in vitamin E, a known scavenger of peroxy and alkoxy radicals [21]. It is likely the ROS induced alterations are localized to these protection mechanisms, and the harmonic response is sensitive to changes in the protection mechanisms e.g., when vitamin E is attacked by ROS , its dipole moment will change and may manifest as a change in harmonic response.

We note that MV also produces ROS, but this production is entirely outside the thylakoid (in contrast to the ROS produced by NH_4_Cl [22], which is produced within the thylakoid membrane and cannot be dismutated via externally applied superoxide dismutase). However, MV by itself does not elicit significant change in ΔV_2f_ as compared to the action of the uncouplers. This may be due to the dilution of ROS outside of the thylakoid by the surrounding buffer. 

Membrane protein phosphorylation may also couple into the NDS measurement. Illuminated thylakoid membrane protein kinase can phosphorylate certain thylakoid polypeptides [23,24,25]. The possibility of phosphorylation increases with the rate of ETC activity, and this phosphorylation will alter the original protein dipole moment. The phosphorylated proteins are located on the outside surface of the thylakoid, and exposure to the surrounding buffer may inhibit dephosphorylation processes. Since such dipole moment alterations are focused near the thylakoid membrane, it is expected that such phosphorylation induced dipole moment alterations could also be detected by NDS.

## 4. Conclusions

Thylakoid membranes provide an ideal system from which to investigate the physics of NDS. Single cell micro-organisms are exceedingly complex, with many regulatory systems that complicate interpretation of NDS experiments. In contrast, thylakoid membranes are relatively simple but provide the essential elements of the ETC. Here, we find photosynthetic ETC activity does not directly generate a strong NDS response, but does indirectly contribute to the 2nd harmonic change, suggesting membrane protein alterations (as opposed to protein pump activity) as the dominant contributor to the NDS signal. The exact protein modifications which couple to ΔV_2f_ are not known, but we speculate that ROS chemistry or membrane phosphorylation may play a role. Further experiments to clarify this point are in preparation. In addition, the choice of electron acceptor system is found to play a role in the reproducibility of the measurements, and, by changing the electrode/electron acceptor system, further improvements of the signal to noise ratio may be possible.
